# The startle disease mutation α1S270T predicts shortening of glycinergic synaptic currents

**DOI:** 10.1113/JP279803

**Published:** 2020-06-18

**Authors:** Zhiyi Wu, Remigijus Lape, Lea Jopp‐Saile, Benjamin J. O'Callaghan, Timo Greiner, Lucia G. Sivilotti

**Affiliations:** ^1^ Department of Neuroscience, Physiology and Pharmacology University College London Gower Street London WC1E 6BT UK

**Keywords:** glycine receptors, human channelopathy, patch‐clamp, single channel recording

## Abstract

**Key points:**

- Loss‐of‐function mutations in proteins found at glycinergic synapses, most commonly in the α1 subunit of the glycine receptor (GlyR), cause the startle disease/hyperekplexia channelopathy in man.
- It was recently proposed that the receptors responsible are presynaptic homomeric GlyRs, rather than postsynaptic heteromeric GlyRs (which mediate glycinergic synaptic transmission), because heteromeric GlyRs are less affected by many startle mutations than homomers.
- We examined the α1 startle mutation S270T, at the extracellular end of the M2 transmembrane helix.
- Recombinant heteromeric GlyRs were less impaired than homomers by this mutation when we measured their response to equilibrium applications of glycine.
- However, currents elicited by synaptic‐like millisecond applications of glycine to outside‐out patches were much shorter (7‐ to 10‐fold) in all mutant receptors, both homomeric and heteromeric. Thus, the synaptic function of heteromeric receptors is likely to be impaired by the mutation.

**Abstract:**

Human startle disease is caused by mutations in glycine receptor (GlyR) subunits or in other proteins associated with glycinergic synapses. Many startle mutations are known, but it is hard to correlate the degree of impairment at molecular level with the severity of symptoms in patients. It was recently proposed that the disease is caused by disruption in the function of presynaptic homomeric GlyRs (rather than postsynaptic heteromeric GlyRs), because homomeric GlyRs are more sensitive to loss‐of‐function mutations than heteromers. Our patch‐clamp recordings from heterologously expressed GlyRs characterised in detail the functional consequences of the α1S270T startle mutation, which is located at the extracellular end of the pore lining M2 transmembrane segment (18ʹ). This mutation profoundly decreased the maximum single‐channel open probability of homomeric GlyRs (to 0.16; cf. 0.99 for wild type) but reduced only marginally that of heteromeric GlyRs (0.96; cf. 0.99 for wild type). However, both heteromeric and homomeric mutant GlyRs became less sensitive to the neurotransmitter glycine. Responses evoked by brief, quasi‐synaptic pulses of glycine onto outside‐out patches were impaired in mutant receptors, as deactivation was approximately 10‐ and 7‐fold faster for homomeric and heteromeric GlyRs, respectively. Our data suggest that the α1S270T mutation is likely to affect the opening step in GlyR activation. The faster decay of synaptic currents mediated by mutant heteromeric GlyRs is expected to reduce charge transfer at the synapse, despite the high equilibrium open probability of these mutant channels.

## Key points

- Loss‐of‐function mutations in proteins found at glycinergic synapses, most commonly in the α1 subunit of the glycine receptor (GlyR), cause the startle disease/hyperekplexia channelopathy in man.
- It was recently proposed that the receptors responsible are presynaptic homomeric GlyRs, rather than postsynaptic heteromeric GlyRs (which mediate glycinergic synaptic transmission), because heteromeric GlyRs are less affected by many startle mutations than homomers.
- We examined the α1 startle mutation S270T, at the extracellular end of the M2 transmembrane helix.
- Recombinant heteromeric GlyRs were less impaired than homomers by this mutation when we measured their response to equilibrium applications of glycine.
- However, currents elicited by synaptic‐like millisecond applications of glycine to outside‐out patches were much shorter (7‐ to 10‐fold) in all mutant receptors, both homomeric and heteromeric. Thus, the synaptic function of heteromeric receptors is likely to be impaired by the mutation.

## Introduction

Glycine receptors mediate fast inhibitory synaptic transmission in the brainstem and spinal cord, where they are essential for motor control (Lynch, 2004). In man, inherited disruption of glycinergic mechanisms produces hyperekplexia or startle disease, a neurological disorder that presents soon after birth with generalised stiffness and exaggerated startle responses to non‐noxious stimuli. The generalised hypertonia fades with age, but the exaggerated startle reflexes, followed by short episodes of hypertonia, persist through life (Bakker *et al*. 2006; Bode & Lynch, 2014). Hyperekplexia can result from loss‐of‐function mutations in most of the key proteins present at glycinergic synapses. These include not only the α1 and β subunits of the glycine receptor (GlyR), which are the constituents of the adult synaptic channel, but also the neuronal glycine transporter (GlyT2) and GlyR anchoring proteins, such as gephyrin and collybistin (Davies *et al*. 2010). Mutations in the GlyR α1 subunit are the most common cause of hyperekplexia, and their widespread location in the protein has highlighted the role of different channel domains in signal transduction (Lynch *et al*. 1997; Lewis *et al*. 1998; Lape *et al*. 2012). Our work on the human startle disease mutant α1K276E (Lape *et al*. 2012) and the *spasmodic* mouse mutant α1A52S (Plested *et al*. 2007) showed that these mutations shorten the time single GlyR channels are active, and therefore the decay of currents produced by fast, quasi‐synaptic glycine applications to outside‐out patches (Wyllie *et al*. 1998). Comparable speeding up of the synaptic current decay has been described for glycinergic currents in spinal slices from mice with the *spasmodic* mutation (Graham *et al*. 2006), and in artificial glycinergic synapses in culture, where the postsynaptic GlyRs are heterologously expressed to have a defined composition (Zhang *et al*. 2015). Despite this agreement between molecular and cellular results, the severity of symptoms in patients or in transgenic mice is but poorly correlated with the degree of loss‐of‐function at receptor level and with the reduction in charge transfer expected at glycinergic synapses. The picture is made more complex by the demonstration that hyperekplexia can be caused also by some (relatively mild) gain‐of‐function mutations in the α1 subunit (Zhang *et al*. 2016).

Recently, it has been hypothesised that the form of GlyR responsible for the symptoms of startle disease is not the adult synaptic receptor, the α1β GlyR heteromer, but the homomeric α1 GlyR (Xiong *et al*. 2014), which is also functional in heterologous expression and occurs presynaptically in the CNS, at locations that include the calyx of Held (Turecek & Trussell, 2001). The proposed role of the homomeric receptor is based on the finding that, for a subset of startle mutations, loss of function is much less pronounced in heteromeric (postsynaptic) GlyRs than in (presynaptic) homomers. In other words, the startle phenotype can often be substantially ‘rescued’ by co‐expression of wild‐type β subunits (Xiong *et al*. 2014). This presynaptic hypothesis of course cannot explain why startle pathology occurs with mutations of other glycinergic synapse proteins that are exclusively postsynaptic, such as the β GlyR subunit and scaffolding proteins.

It is important to note that, of the many startle mutations identified, only a handful have been characterised in detail in synaptic‐like conditions, such as glycinergic synapses in culture (Zhang *et al*. 2015), or with fast Gly applications (Lape *et al*. 2012). There is a clear need of more information, especially on mutations that can be rescued by β subunit expression (a feature that has not been systematically investigated since the original report). Here we present the first detailed functional characterisation of α1S270T, a hyperekplexia mutation that causes a dominant form of startle disease (Lapunzina *et al*. 2003). We chose this mutation because it is one of those whose function has been reported to be rescued by β subunit expression (Xiong *et al*. 2014).

In our experiments, we found that α1S270T reduced macroscopic GlyR sensitivity to glycine to a similar extent in homomeric and heteromeric GlyRs. At single channel level, maximum open probability was reduced by almost 5‐fold only in homomers. In heteromers, a high open probability was reached despite the mutation, but it required very high glycine concentrations, 30‐fold greater than those likely to be reached at the synapse. Loss of gating function manifested itself also in the faster decline of GlyR currents activated by millisecond, quasi‐synaptic application of high glycine concentrations. This faster decay was similar in homomeric and heteromeric GlyRs.

## Methods

### Cell culture and transfection

Human embryonic kidney 293A cells (HEK293A; ATCC, Teddington, UK, Cat. No. PTA‐4488, RRID:CVCL0045) were used for transient expression of wild‐type or mutant homomeric α1 or heteromeric α1β GlyRs. The S270T hyperekplexia missense mutation was introduced into the human α1 GlyR subunit using the QuickChange site‐directed mutagenesis kit (Agilent Technologies, Santa Clara, CA, USA, Cat. No. 200514). The entire open reading frame of the plasmids was sequenced by Source Bioscience (Cambridge, UK) to confirm successful mutation incorporation.

HEK293A cells were cultured in 25 cm^2^ vented culture flasks containing 5 ml of Dulbecco's modified Eagle's medium (DMEM; Gibco Thermo Fisher, Loughborough, UK, Cat. No. 41966‐029) supplemented with fetal bovine serum (Gibco Cat. No. 10500‐064), non‐essential amino acids (Gibco Cat. No. 11140‐050) and penicillin/streptomycin (Gibco Cat. No. 15140‐122). Cells were maintained at 37°C, 95% air/5% CO_2_ in a humidified incubator and passaged every 2–3 days in order to maintain ∼70% confluence.

HEK293A cells were transiently transfected using the calcium phosphate‐DNA precipitation method. The DNA mixture consisted of pcDNA3.1 plasmids with inserts coding for the enhanced green fluorescence protein (eGFP; GenBank accession number U55763.1), and the human glycine receptor subunits α1 (wild type or bearing the S270T mutation) with or without the β subunit (GenBank accession numbers P23415.2 or P48167.1 for wild‐type α1 and β, respectively). Plasmid without an open reading frame (‘empty’) was added to the transfection mixture to optimise the expression level (Groot‐Kormelink *et al*. 2002). In each case we used a total of 6 μg of cDNA per dish. For wild‐type homomeric receptors, the final mixture of cDNA contained 2% α1, 18% eEGFP and 80% empty pcDNA3.1 plasmid. For mutant homomeric receptors, the proportion of empty plasmid was reduced to 62% and the proportion of α1S270T subunit plasmid increased to 20%. In order to express heteromeric receptors (wild‐type or mutant), the cDNA mixture contained 2% α1, 80% β and 18% eGFP plasmids. Cells were washed 5–16 h later and electrophysiological experiments were performed 1–2 days after transfection.

### Patch‐clamp recordings and analysis

Macroscopic and single‐channel currents were recorded from transfected HEK293A cells in the whole‐cell, outside‐out and cell‐attached patch‐clamp configurations at 20°C. Transfected cells were visualised using an inverted fluorescence microscope (Olympus IX71, Olympus UK, Southend‐on‐Sea, UK). Patch electrodes were pulled from thick‐walled borosilicate glass capillary tubes (GC150F‐7.5; Harvard Apparatus UK, Cambourne, UK) on a Flaming/Brown puller (Model‐P97, Sutter Instruments, Novato, CA, USA). Pipettes for single‐channel recordings were coated near the tip with Sylgard 184 (Dow Corning, Dow Silicones, Dewsbury, UK). Electrodes were fire‐polished at a microforge to give a final resistance of 3–5 MΩ for whole‐cell recordings and 5–15 MΩ for cell‐attached or outside‐out recordings (when filled with the appropriate solution).

Cells were bathed in an extracellular solution consisting of (in mM): sodium d‐gluconate (20), NaCl (112.7), KCl (2), CaCl_2_ (2), MgCl_2_ (1.2), hepes (4‐(2‐hydroxyethyl)‐1‐piperazineethanesulfonic acid; 10), glucose (40) (all Sigma‐Aldrich, Gillingham, UK) and tetraethylammonium chloride (TEACl;10) (Alfa Aesar, Thermo Fisher, Cat. No. A15215) adjusted to pH 7.4 with NaOH (osmolarity ∼320 mOsm/l). To record macroscopic currents the pipette was filled with a 30 mm chloride intracellular solution, containing (in mm): potassium gluconate (101.1), ethylene glycol tetraacetic acid (EGTA; 11), CaCl_2_ (1), MgCl_2_ (1), hepes (10), MgATP (2), sucrose (40), KCl (6) (all Sigma) and TEACl (20), adjusted to pH 7.4 with NaOH (osmolarity ∼320 mOsm/l). The pipettes for cell‐attached recordings were filled with extracellular solution containing the required concentration of agonist. All solutions were prepared in bi‐distilled water or, for cell‐attached single channel recordings, in high‐performance liquid chromatography grade (HPLC) water (VWR Chemicals, Lutterworth, UK), and filtered through a 0.2 μm cellulose nitrate membrane filter (Whatman, GE Healthcare, Maidstone, UK, Cat. No. 7182‐004) before use. Glycine or sarcosine‐containing solutions were prepared by diluting a 1 m glycine (Sigma‐Aldrich, Cat. No. 50049) or 1 m sarcosine (Sigma‐Aldrich, Cat. No. S7672) stock in extracellular solution.

Sarcosine was tested at 100 mm for glycine contamination by an HPLC assay: samples were resuspended in 10 μl 50% EtOH, reacted for 30 min with 90% EtOH:triethylamine:phenylisothiocyanate (7:2:1), evaporated to dryness at room temperature and redissolved in 100 μl 5% acetonitrile in 0.1 m ammonium acetate. A 150 × 4.6 mm Hypersil C18 column was used. Molecules of interest were detected at 254 nm. Sarcosine was found to be contaminated by about 10 μm glycine per 100 mm. Sarcosine (approx. 7 g) was therefore purified by re‐crystallising it three times from 95% ethanol, obtaining 2.37 g of purified compound, whose ^1^H‐NMR and elemental analysis were consistent with those expected for sarcosine. The glycine contamination of a 100 mm solution of purified sarcosine was below the detection limit of the HPLC assay (*ca* 1 μm). Only purified sarcosine was used in the single channel experiments.

### Whole‐cell recording

Whole‐cell currents were recorded from isolated transfected cells held at a holding potential of −60 mV (corrected for the junction potential of 11 mV) with an Axopatch 200B amplifier (Molecular Devices, San Jose, CA, USA) and filtered at 5 kHz by the amplifier's low pass 4‐pole Bessel filter. Recordings were digitised with a Digidata 1440A (Molecular Devices) at a sampling frequency of 20 kHz and acquired to PC using Clampex 10.2 software (Molecular Devices; RRID:SCR_011323) for offline analysis. Access resistance was below 10 MΩ and was compensated by at least 70%.

Agonist solutions were applied to the cells for approximately 1–2 s by a U‐tube application system (Krishtal & Pidoplichko, 1980), with a 10–90% exchange time <1 ms (as tested by application of 50% diluted extracellular solution to the open tip pipette). A saturating test dose of glycine was applied at the beginning of each recording and repeated until a stable peak response was observed. This saturating agonist concentration was applied also every third or fourth application to check response stability throughout the recording. Different agonist concentrations were applied in random order to obtain concentration‐response curves.

The peak current amplitudes for each glycine application were measured using Clampfit 10.2 software (Molecular Devices; RRID:SCR_011323). Response stability was analysed by plotting the current amplitudes of the saturating test doses against time. Only cells where the test response rundown was less than 30% during the experiment were accepted for further analysis. No correction for rundown was applied. The concentration‐response data for each cell were then fitted with the Hill equation using the CVFIT programme (https://github.com/DCPROGS/CVFIT/releases/tag/v1.0.0-alpha) to estimate EC_50_ (agonist concentration required to elicit 50% maximal response), *n*_H_ (Hill‐slope) and *I*_max_ (maximum peak current). The responses for each cell were then normalised relative to their respective fitted *I*_max_ and pooled for display of the concentration‐response curves in the figures.

### Fast agonist application

Macroscopic currents evoked in outside‐out patches by fast agonist application pulses were recorded at a pipette holding potential of −100 mV (−111 mV when corrected for a junction potential of 11 mV). The internal solution was the same as the one for whole‐cell recordings and contained 30 mM chloride. Internal chloride concentration has a profound effect on the kinetics of GlyRs (Pitt *et al*. 2008; Moroni *et al*. 2011). The value of 30 mm was chosen as a compromise between matching the physiological chloride concentration of a few millimolar and maintaining a large current signal. Glycine, dissolved in extracellular solution, was applied to outside‐out patches with a theta tube (Hilgenberg GmbH, Masfeld, Germany, Cat. No. 1407201), cut to a final diameter ≈ 150 μm at the tip. The tube was driven by a piezo stepper PZ‐150M (Burleigh Instruments Inc., Harpenden, UK). The exchange time was measured by the application of diluted bath solution (e.g. 30:70 bath solution:water) before the experiment (to optimise the electrode position) and after the rupture of the patch. The rise and decay times for these open‐tip currents were measured using Clampfit 10.2 as the times from 20 to 80% of the peak response. Patches in which the open tip response had a 20–80% exchange time slower than 200 μs were rejected from further analysis.

In order to study the kinetics of macroscopic currents, 10–20 responses were recorded in response to pulses of glycine applied at intervals of at least 10 s and averaged. Only experiments in which the rundown between the first and last three responses was <30% were included in the analysis. The time course of the macroscopic currents was characterised by fitting the risetime between 20 and 100% and the decay time from 80 to 20% of the peak response with one or more exponentials.

### Single‐channel recording

Low‐noise single‐channel currents were recorded in the cell‐attached configuration at a pipette holding potential of +100 mV with an Axopatch 200B amplifier and filtered at 10 kHz using the amplifier's low pass 4‐pole Bessel filter. The data were digitised with a Digidata 1322A (Molecular Devices) at a sampling frequency of 100 kHz and acquired to PC using Clampex 10.2 for offline analysis.

### Single channel current amplitude and cluster *P*_open_ measurements

In order to compare single‐channel current amplitudes and cluster open probability, single‐channel recordings were filtered offline using the Clampfit 10.2 low‐pass Gaussian filter with a final cut‐off of 5 kHz and resampled at 50 kHz. At high agonist concentration, channel openings occurred in clusters delimited by long closed intervals, likely to be desensitised. These clusters are likely to originate from the activity of a single ion channel molecule and were used for *P*_open_ measurements. Clusters longer than 100 ms with more than 10 openings were selected for analysis. The gap between clusters was at least 300 ms for the homomeric α1S270T GlyR and at least 100 ms for the heteromeric α1S270Tβ GlyR and both wild‐type receptors. Channel activity in the selected clusters was idealised using the half‐amplitude threshold method implemented in Clampfit 10.2 and open probability was calculated as the ratio of cluster open time over total cluster length. The amplitude of single channel currents was measured in Clampfit 10.2 as the average of all detected opening amplitudes inside a cluster.

### Statistics

Results are reported as the mean ± standard deviation (SD), where *n* represents number of cells, clusters or patches as indicated. Non‐parametric randomisation test (two‐tail, non‐paired; 50,000 iterations) was used to determine *P* values for the difference being greater than or equal to the observed difference (DC‐Stats software: https://github.com/DCPROGS/DCSTATS/releases/tag/v.0.3.1-alpha). Where appropriate, the 95% confidence intervals for the differences between means are given.

For the fit of the Hill equation to *P*_open_ cluster data, a single set of data was fitted, thus we report result as fit estimate ± approximate standard deviation estimated from the covariance matrix and two‐unit likelihood intervals for each of the fitted parameters (CVFIT software: https://github.com/DCPROGS/CVFIT/releases/tag/v1.0.0-alpha).

## Results

### Whole‐cell concentration‐response curves

**Figure 1:**
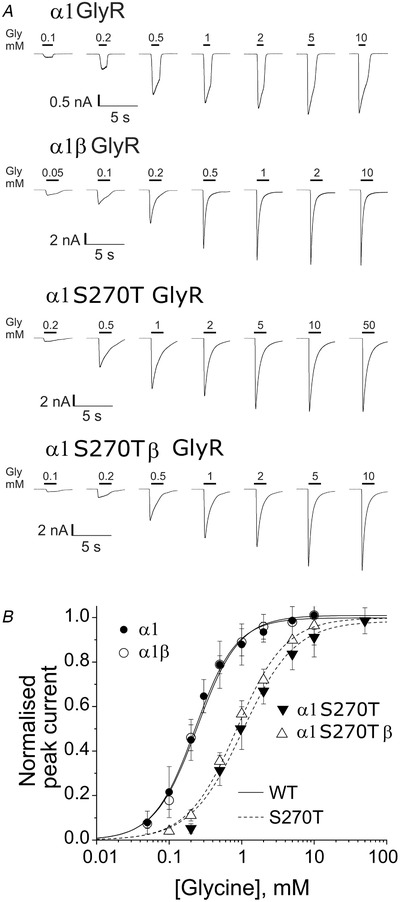
The α1 S270T startle disease mutation reduces the glycine sensitivity of both homomeric and heteromeric GlyR *A*, representative whole‐cell current responses evoked by U‐tube glycine application to HEK293 cells expressing wild‐type or α1S270T mutant homomeric or heteromeric glycine receptors. Bars above the traces show the timing of glycine applications. Currents were recorded with pipettes filled with low‐Cl^−^ solution (30 mm). *B*, the glycine concentration‐response relation for wild‐type (circles) or α1S270T mutant (triangles) glycine receptors (*n* = 5 to 10 cells). Lines are fits of the Hill equation to the data (see Table 1). Curves and data points are normalised to the fitted maxima for each curve.

Figure 1*A* shows the whole‐cell current responses obtained when glycine (0.05–50 mm) was applied by U‐tube to HEK293 cells expressing wild‐type or mutant α1S270T GlyRs. The traces show that these inward currents activated and desensitised quickly during the application, especially at the highest concentrations. The graphs in Fig. 1*B* are the concentration‐response curves plotted from the peak amplitude of the agonist responses, normalised to the fitted maximum of the glycine curve for each receptor form. The glycine EC_50_ values estimated from fitting the Hill equation to the data points were very similar for wild‐type homomeric and heteromeric receptors: 0.24 ± 0.06 mm (*n *= 10 cells, see Table 1) and 0.23 ± 0.03 mm (*n *= 5 cells), respectively. The α1S270T mutation increased the glycine EC_50_ in both homomeric and heteromeric GlyRs, by 4.5‐ and 3.7‐fold (to 1.1 ± 0.3 and 0.9 ± 0.1 mm, *n* = 8 cells; *P *= 0.00002 and 0.0006, respectively). Thus, the glycine sensitivity of the mutant heteromeric receptor was not restored to wild‐type values by the co‐expression of the β subunit (Table 1).

**Table 1:** Effects of the α1S270T startle disease mutation on the whole‐cell concentration‐response curves of recombinant homomeric and heteromeric GlyR

Agonist, receptor	*I*_max_ (nA)	EC_50_ (mM)	*n*_H_	*I*_agonist_/*I*_Gly_	*n*
Glycine, WT α1	7 ± 5	0.24 ± 0.06	1.6 ± 0.2	1	10
Glycine, WT α1β	5 ± 5	0.23 ± 0.03	1.7 ± 0.2	1	5
Glycine, α1S270T	13 ± 2	1.1 ± 0.3	1.1 ± 0.2	1	8
Glycine, α1S270Tβ	7 ± 6	0.9 ± 0.1	1.29 ± 0.08	1	8
Sarcosine, WT α1	5 ± 2	14 ± 6	1.5 ± 0.1	0.6 ± 0.2	7
Sarcosine, α1S270T	0.8 ± 0.4	23 ± 3	1.4 ± 0.3	0.08 ± 0.04	6

Hill equation fits to agonist concentration‐response data from whole‐cell recordings; holding potential −60 mV, internal chloride 30 mm.

### Cell‐attached single‐channel recordings at saturating agonist concentration

**Figure 2:**
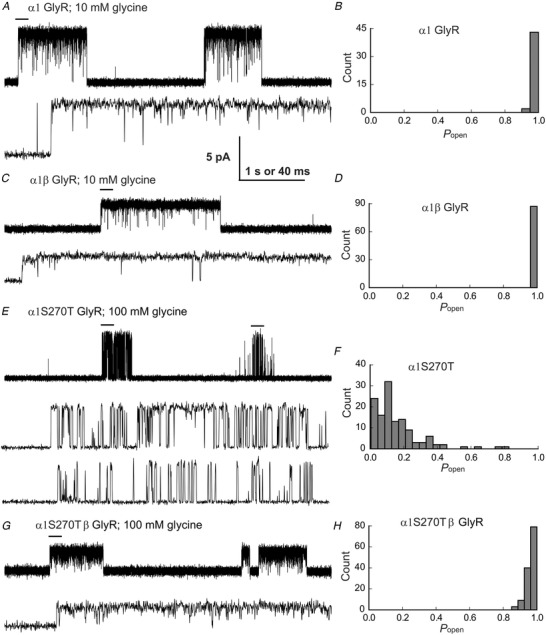
The α1S270T startle disease mutation reduces the maximum single‐channel open probability in homomeric glycine receptors, but not in heteromers Cell‐attached single‐channel currents recorded at saturating glycine concentration. *A*, *C*, *E* and *G*, continuous 5 s records (top traces in each panel) and 0.2 s sweeps (lower traces in each panel are expanded stretches indicated by bars in 5 s traces) from wild‐type α1 (*A*), wild‐type α1β (*C*), mutant α1S270T (*E*) and mutant α1S270Tβ (*G*) receptors at saturating glycine concentrations. The traces show clearly the decrease in maximum *P*_open_ in homomeric GlyR (*E*). *B*, *D*, *F* and *H*, histograms of cluster *P*_open_ for each receptor. Note the wide spread of the *P*_open_ values of homomeric α1S270T receptor clusters (*F*).

Impairment in gating is the most common phenotype associated with startle disease mutations. Direct measurement of the maximum single‐channel open probability, *P*_open_, elicited by high agonist concentrations is a sensitive and reliable test of whether a mutation has affected channel gating (Colquhoun, 1998). The traces in the left‐hand panels of Fig. 2 are typical examples of cell‐attached single‐channel activity at saturating glycine concentrations. At 10 mm glycine, openings of wild‐type α1 or α1β GlyRs (Fig. 2*A* and *C*, respectively) occurred in clusters, flanked by long periods of inactivity likely due to desensitisation. The traces show also that the amplitude of homomeric openings was higher than that of heteromeric GlyR (5.6 ± 0.5 *vs*. 2.5 ± 0.2 pA, respectively; *P* << 10^−6^, two‐tailed randomisation test), as expected, given the higher conductance of homomeric receptors (Bormann *et al*. 1993).

The average maximum cluster *P*_open_ was high, with very little variability, for both homomeric and heteromeric receptors, as shown by the histograms in Fig. 2*B* and *D* (0.99 ± 0.02 and 0.993 ± 0.006, *n* = 45 clusters from 4 patches and 87 clusters from 4 patches respectively; Table 2).

**Table 2:** Effects of the α1S270T startle disease mutation on the properties of single‐channel clusters evoked by saturating concentrations of agonist in cell‐attached recordings

	*P*_open_ (range)	Amplitude (pA)	Duration (s)	*n* (clusters/ patches)
Glycine10 mm, WT α1	0.99 ± 0.02 (0.90–0.999)	5.6 ± 0.5	0.6 ± 0.5	45/4
Glycine10 mm, WT α1β	0.993 ± 0.006 (0.96–1.0)	2.5 ± 0.2	2 ± 2	87/5
Glycine100 mm, α1S270T	0.16 ± 0.1 (0.005–0.83)	4.4 ± 0.8	1 ± 1	128/10
Glycine 100 mm, α1S270Tβ	0.96 ± 0.03 (0.85–1.0)	2.9 ± 0.6	0.6 ± 0.6	131/4
Sarcosine 100 mm, WT α1	0.58 ± 0.2 (0.29–0.94)	4.3 ± 0.5	0.6 ± 0.4	21/1
Sarcosine100 mm, α1S270T	<0.01^*^	3.9 ± 0.5	—	0/3

* No clusters were observed in α1S270T GlyR single channel records in the presence of 100 mM sarcosine, thus *nP*_open_ was measured and is presented in the table.

For mutant GlyRs, we chose 100 mm as a saturating glycine concentration, on the basis of the whole‐cell concentration‐response curves in Fig. 1*B*. The traces in Fig. 2*E* show that openings of homomeric α1S270T GlyR also occurred in clusters, but the appearance of the clusters was clearly different. In the homomeric mutant, the maximum *P*_open_ was much lower (0.16 ± 0.1, *n* = 128 clusters from 10 patches; *P* << 10^−6^, two‐tailed randomisation test) than in wild‐type receptors. Furthermore, the cluster appearance was much less consistent, with a wide spread of *P*_open_ values (range 0.005–0.826) as shown in the histogram in Fig. 2*F*. This is clearly visible in the two clusters shown in the top trace of Fig. 2*E*, where the cluster on the left has a *P*_open_ of 0.54 and the one on the right 0.06 (200 ms of each cluster marked by a bar are shown also at faster time scale in Fig. 2*E*, middle and bottom traces).

We next examined the effect of the mutation on the heteromeric channel, which is the form of GlyR that is relevant to adult synaptic transmission. Typical clusters of single channel openings of mutant heteromeric GlyR at 100 mm glycine are shown in Fig. 2*G* (top trace). The open probability of these clusters was much higher than for the mutant homomer (0.96 ± 0.03; *n* = 131 clusters from 4 patches; *P* << 10^−6^, two‐tailed randomisation test), and very close to the wild‐type value (see also Fig. 2*H*), although with much higher cluster‐to‐cluster variability (compare Fig. 2*D* and *H*). Thus, the β subunit does rescue the receptor maximum *P*_open_ to a value similar to that of wild type, but this value was reached at a higher glycine concentration (Fig. 1*B*).

### The partial agonist sarcosine

**Figure 3:**
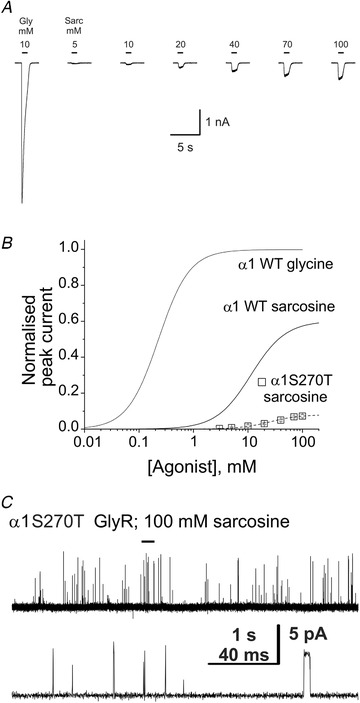
The α1S270T startle disease mutation reduces the potency and the efficacy of the partial agonist sarcosine in homomeric GlyR *A*, representative whole‐cell current responses evoked by U‐tube sarcosine application to HEK293 cell expressing wild‐type or α1S270T mutant homomeric GlyR. Bars above the traces show the timing of agonist applications. The first trace shows the response to 10 mm glycine used to normalise the concentration‐response curve in this cell. Currents were recorded with pipettes filled with low‐Cl^−^ solution (30 mm). *B*, the sarcosine concentration‐response relation for homomeric wild‐type or α1S270T mutant glycine receptors. Symbols represent average data for *n* = 6–7 cells. The lines are the fits of the Hill equation to the data (see Table 1). Curves and data points are normalised to the maximum glycine‐activated current in each cell. Note the marked reduction in the maximum response to sarcosine (relative to glycine) in α1S270T homomeric receptors. *C*, traces show typical single channel openings produced by 100 mm sarcosine in mutant homomeric GlyR, recorded in the cell‐attached mode. Note that sarcosine failed to evoke distinguishable clusters of openings, and channel openings were sparse and brief.

Because of the heterogeneity in the single channel activity of the mutant, we felt it useful to confirm that gating is impaired in the whole population of α1S270T mutant GlyRs. It is not possible to detect this effect simply by comparing the absolute size of maximum currents in whole‐cell experiments, because we do not know how many channels are on the cell surface. The robust way to prove by a macroscopic experiment that gating is impaired in the whole channel population is to show that the maximum response to a partial agonist, relative to that of the full agonist glycine in the same cell, is reduced by the mutation. We therefore obtained whole‐cell concentration‐response curves for sarcosine (*N*‐methyl‐glycine) (Zhang *et al*. 2009), which is a partial agonist in homomeric wild‐type GlyRs (Safar *et al*. 2017). Panels *A* and *B* of Fig. 3 show that the S270T mutation reduced both the potency of sarcosine (from 14 ± 6 mm to 23 ± 3 mm; *n* = 6 cells; *P* = 0.0076, two‐tailed randomisation test) and its maximum response relative to glycine (from 60 ± 20% to 8 ± 4%; *P* = 0.00014, two‐tailed randomisation test). This marked reduction in sarcosine maximum (7.5‐fold) confirms that the α1S270T mutation impairs channel gating.

In wild‐type homomeric α1 GlyRs, a near‐saturating concentration of sarcosine (100 mm) elicits clusters of openings, with a mean *P*_open_ of 0.58 ± 0.2 (Safar *et al*. 2017).

Figure 3*C* shows activity evoked by 100 mm sarcosine in α1S270T homomeric GlyRs. Mutant homomeric channels displayed only sparse openings in response to 100 mm sarcosine and no clusters could be detected (*n* = 3 patches). Channel openings were generally very short in duration and mostly failed to reach full amplitude. Measurement of the few fully resolved longer openings present gave an amplitude of 3.9 ± 0.5 pA (*n* = 11 openings from 3 patches). Since no clusters could be identified in these recordings, we measured the *nP*_open_ of the entire record at 100 mm sarcosine and estimated its value to be approximately 0.01 (Table 2).

### Concentration dependence of homomeric α1S270T GlyR single‐channel activity

**Figure 4:**
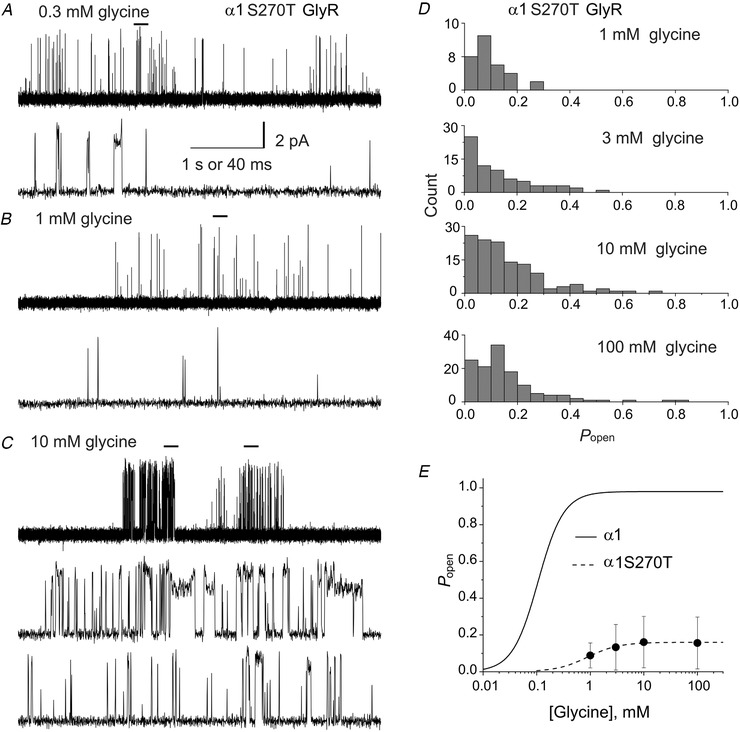
Concentration dependence of the open probability of homomeric α1S270T glycine receptor clusters *A*–*C*, examples of continuous 5 s records (top traces in each panel) at 0.3 (*A*), 1 (*B*) and 10 mm (*C*) glycine. The bars over the top traces indicate the regions expanded in time and depicted in the lower traces in each panel. *D*, histograms of open probabilities of clusters recorded at 1, 3, 10 and 100 mm glycine. *P*_open_ values were widely spread at all concentration. *P*_open_ values were obtained for each patch from the ratio between the total open time and the total duration of the clusters, from recordings idealised by half‐amplitude threshold crossing. *E*, cluster open probability plot *vs*. glycine concentration. The filled circles are the mean values (error bars for the SD of the mean are smaller than the symbols) for the patches included in the analysis (33–128 clusters from 2–4 patches at each concentration). The dashed line is a fit of the Hill equation to the homomeric α1S270T receptor data and the continuous line is the concentration *P*_open_‐ curve for wild‐type homomeric receptor from (Beato *et al*. 2004).

In order to obtain a robust confirmation of the effects of the mutant on agonist potency *vs*. efficacy, we obtained single‐channel recordings of homomeric mutant GlyRs in the presence of 0.3, 1 and 10 mm glycine (Fig. 4*A*–*C*). At low glycine concentrations (up to 0.3 mm), individual channel activations were sparse and occurred as single openings, or bursts of few openings as shown in Fig. 4*A* (top and bottom traces at slower and faster time scales, respectively). The longer shut times in these records were concentration dependent, and therefore likely to reflect primarily long sojourn(s) in un‐liganded states (rather than desensitisation). At this low concentration we could not measure single channel open probability because we could not identify stretches of openings and shuttings that reflect the activity of one channel molecule.

At glycine concentrations equal to or higher than 1 mm, the mutant receptors enter into long‐lived desensitised states (Sakmann *et al*. 1980; Beato *et al*. 2004), which manifest as concentration‐independent shut times between clusters of channel activations (in wild‐type receptors clustering was observed from 50 μm, Beato *et al*. 2004). Each cluster reflects the activity of a single GlyR molecule that emerges for a time from desensitisation and this allows us to measure single channel *P*_open_ in the cluster. We observed substantial variability in the *P*_open_ at every concentration, as expected from the variability seen at saturating glycine concentrations (Fig. 2*E* and *F*). Nevertheless, the effect of increasing glycine concentration was clear. Figure 4*B* depicts the first 4 s of a long cluster elicited by the lowest cluster concentration (1 mm glycine). This cluster was preceded by a long shut interval of 31 s (only ∼1 s of which are shown here). At higher agonist concentration (10 mm glycine), the clusters are shorter and their variability more obvious, as shown by Fig. 4*C*.

The three clusters in Fig. 4*C* are separated by shuttings longer than 100 ms and have *P*_open_ of 0.28, 0.02 and 0.06. Two stretches from the highest and lowest *P*_open_ clusters are depicted at a slower time scale (Fig. 4*C*, middle and bottom traces). Higher *P*_open_ clusters were different, because they had shorter intracluster shut intervals and longer open times. This was reflected in a strong positive correlation between cluster *P*_open_ and cluster mean open time (e.g. *r* = 0.68 for clusters in 10 mm glycine). In addition to that, many long (1–10 ms) sojourns in a lower conductance level were detectable.

The histograms in Fig. 4*D* show the wide scatter of for the cluster *P*_open_ values at all glycine concentrations (1–10 mm). Plotting the mean cluster *P*_open_ values gives a concentration‐response curve with a shallow concentration dependence (Fig. 4*E*; cf. the reference *P*_open_ curve for the wild type, Beato *et al*. 2004).

The fit with a Hill equation (dashed line) gave a maximum *P*_open_ of 0.16 ± 0.01 (2‐unit likelihood interval 0.14–0.22), EC_50_ of 0.9 ± 0.4 mm (2‐unit likelihood interval n/a – 1.7 mm) and Hill slope of 1.5 ± 0.8 (2‐unit likelihood interval 0.2 – n/a; Table 3). Note that EC_50_ and Hill slope were poorly defined, probably because we could not identify clusters at concentrations lower than 1 mm. With these limitations, the increase in glycine EC_50_ produced by the mutation was similar in the single channel and in the whole‐cell‐experiments (approximately 8.5‐fold and 4.5‐fold, respectively).

**Table 3:** Effects of the α1S270T startle disease mutation on the concentration dependence of single channel *P*_open_ of glycine receptors

	Maximal *P*_open_	EC_50_ (μM)	*n*_H_	*n* (clusters at each concentration)
α1 GlyR WT	0.98	106	1.8	—
α1β GlyR WT	0.97	60	3.4	—
α1S270T GlyR	0.16 ± 0.01	900 ± 400	1.5 ± 0.8	range: 33–128
α1S270Tβ GlyR	0.91 ± 0.02	320 ± 30	1.1 ± 0.1	range: 41–131

Data for wild‐type receptors are for rat α1 and α1β GlyR, from Beato *et al*. 2004 and from Burzomato *et al*. 2004, respectively. Data for mutant receptors are shown as mean ± approximate standard deviation calculated from the covariance matrix.

**Table 4:** Effects of the α1S270T startle disease mutation on the activation and deactivation time course of glycine currents

	Peak current (pA)	Activation τ (ms)	Deactivation τ (ms)	*n*
WT α1	5000 ± 4000	0.13 ± 0.04	12 ± 5	6
WT α1β	110 ± 80	0.2 ± 0.1	10 ± 6	5
α1S270T	60 ± 30	0.4 ± 0.1	1.2 ± 0.3	6
α1S270Tβ	200 ± 200	0.18 ± 0.03	1.5 ± 0.6	7

Experimental values measured from currents elicited by the fast application of 2 ms pulses of 3 mm glycine to outside‐out patches. *V*_holding_ −111 mV; 30 mm internal chloride; open tip exchange time better than 200 μs.

### Concentration dependence of heteromeric α1S270Tβ GlyR single‐channel activity

**Figure 5:**
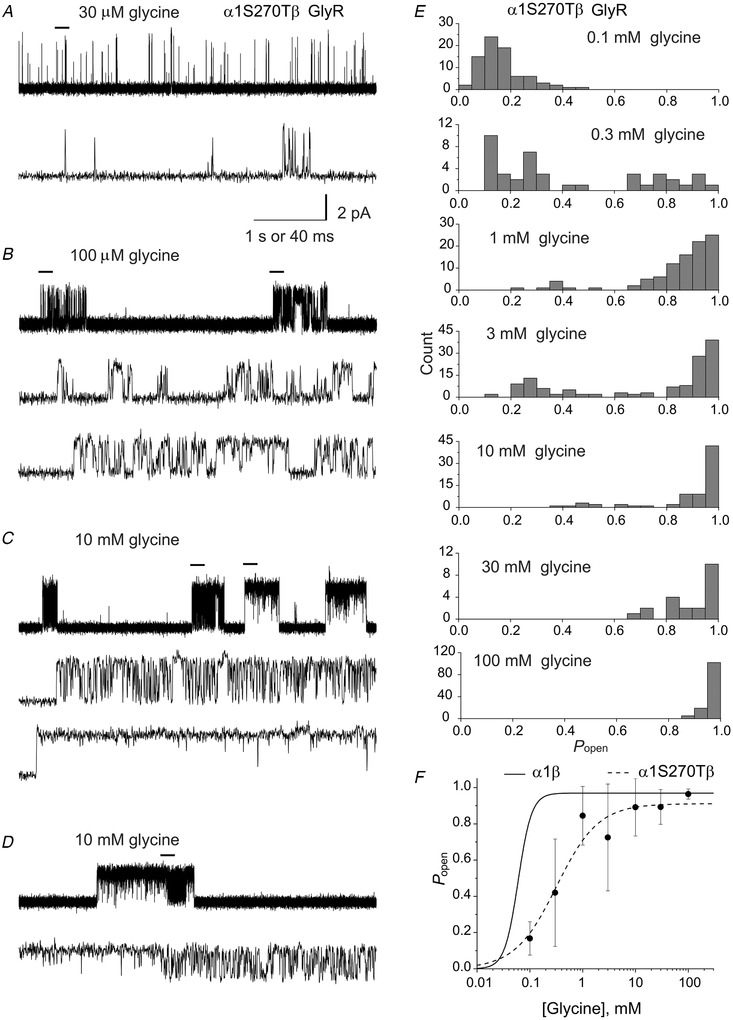
Concentration dependence of the open probability of heteromeric α1S270Tβ glycine receptor clusters *A*–*D*, examples of continuous 5 s records (top traces in each panel) at 0.03 (*A*), 0.1 (*B*) and 10 mm (*C* and *D*) glycine. The bars over the top traces indicated the regions expanded in time and depicted as lower traces in each panel. *E*, histograms of open probabilities of clusters recorded at 0.1, 0.3, 1, 3, 10, 30 and 100 mm glycine. *P*_open_ values were widely spread at all concentrations. *F*, cluster open probability plot *vs* glycine concentration. The filled circles are the mean values of all the analysed clusters (*n *= 21–131). The continuous line is the concentration open probability curve for heteromeric α1β channels from (Burzomato *et al*. 2004).

We repeated the same single channel experiments for the heteromeric α1S270Tβ GlyR, obtaining cell‐attached recordings in the presence of 10 μm to 100 mm glycine (Fig. 5).

At the lowest glycine concentrations of 10 μm (data not shown) and 30 μm (Fig. 5*A*), the α1S270Tβ GlyR activations appeared as single openings or bursts of openings (see the second, expanded trace) and clusters could not be identified (note that, in wild‐type heteromeric GlyR, clear clustering was observed at 30 μm, Burzomato *et al*. 2004). The expanded stretch of record shows also that the majority of openings were very short and rarely reached full amplitude.

At higher glycine concentrations (≥100 μm) channel activity appeared as clusters of openings separated by long shut intervals lasting hundreds of milliseconds or seconds (Fig. 5*B* and *C*). Cluster‐to‐cluster variability in open probability was seen at most concentrations, but was more prominent at the lower ones (see Fig. 5*E*). The top trace in Fig. 5*B* (top trace) shows two clusters recorded at 100 μm glycine. Stretches for each of these clusters are shown in Fig. 5*B* (middle and bottom traces) at a faster time scale. The first cluster was formed by shorter openings and longer shuttings than those in the second cluster. The *P*_open_ values of the first and the second cluster were 0.195 and 0.493, respectively, and the average *P*_open_ at 100 μm glycine was 0.18 ± 0.09 for 81 clusters from 5 patches.

Even at 10 mm glycine, a concentration near the maximum of the macroscopic dose‐response curve, clusters with different behaviour were obvious (see Fig. 5*C*). These clusters belonged to two distinct types. The first two clusters in the trace in Fig. 5*C* (top) had low *P*_open_ values (0.591 and 0.684), whereas the last two had a *P*_open_ values of 0.996 and 0.989. It was not clear whether the different clusters came from different populations of channels or represented different gating modes of the same molecule. The latter seems more likely, as both patterns were observed in same patch and in a few cases both patterns appeared in the same cluster (as shown by the traces at 100 mm in Fig. 5*D*).

Figure 5*E* shows the distribution of *P*_open_ values for all heteromeric α1S270Tβ GlyR clusters. At lower concentrations, the spread of *P*_open_ values was particularly high, ranging from 0.125 to 0.993 at 3 mm glycine. As the agonist concentration increased, the proportion of low *P*_open_ clusters decreased, disappearing at 100 mm. At 10 mm glycine, the majority of clusters (62 out of 73) had *P*_open_ values higher than 0.8 (average 0.95 ± 0.05), and only 11 clusters had *P*_open_ values lower than 0.8 (average 0.58 ± 0.1). The overall pattern is clearly different from that observed in homomeric GlyRs. Despite the large number of clusters analysed (between 21 and 131 for each concentration), there were no clearly defined peaks, and it was not possible to fit the distribution to identify Gaussian components.

Using all the clusters collected gave rise to the concentration‐*P*_open_ curve shown in Fig. 5*F*, where the data points for the mutant (filled circles) fitted by a Hill equation (dashed line) are shown together with a reference concentration‐response curve for the wild‐type heteromeric receptor (continuous line). The mutant curve reached a fitted maximum *P*_open_ similar to that of wild‐type GlyRs (0.91 ± 0.02; 2‐unit likelihood interval 0.85–0.94), but had a shallow Hill slope (1.1 ± 0.1; 2‐unit likelihood interval 0.97–1.9) and a higher EC_50_ (320 ± 30 μm; 2‐unit likelihood interval 230–360 μm).

### Glycine concentration jumps

During synaptic transmission, postsynaptic receptors are exposed to high concentrations of glycine (2–3.5 mm) for less than 1 ms (Beato, 2008). In order to simulate such conditions, we recorded GlyR macroscopic currents elicited by the fast application of short glycine pulses by a theta tube to outside‐out patches. The aim of these recordings was to assess how the α1S270T mutation changes the time course of current deactivation in quasi‐synaptic conditions.

**Figure 6:**
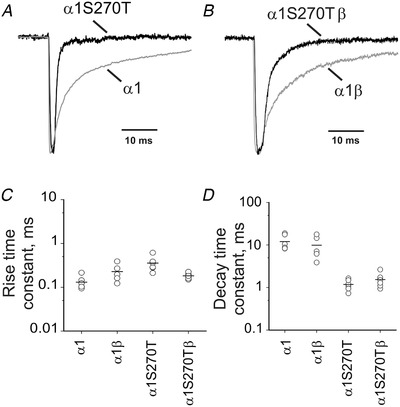
Glycine receptors carrying the α1S270T startle disease mutation deactivate faster in response to synaptic‐like applications of 3 mm glycine *A* and *B*, representative current responses evoked by the fast application of 3 mm glycine (2 ms pulses) via piezo driven θ‐tube to outside‐out patches. Responses by wild‐type (grey trace) and α1S270T (black trace) receptors are shown for homomeric (*A*) and heteromeric (*B*) GlyRs and currents are normalised to their own peak. The internal solution contained 30 mm chloride. The activation of wild‐type GlyRs is fast, and its speed is limited by the solution exchange time. At the end of the glycine pulse, channel deactivation was much faster for both types of mutant receptors than for wild‐type ones. *C* and *D*, the activation (*C*) and deactivation (*D*) time constants for the outside‐out currents evoked by fast application of glycine. The values for each patch are shown as open circles and the mean for each receptor is indicated as a bar.

The traces in the top panels of Fig. 6 are examples of GlyR macroscopic currents recorded from outside‐out patches exposed to 2 ms pulses of 3 mm glycine. Both homomeric and heteromeric wild‐type GlyRs (grey traces in Fig. 2*A* and *B*, respectively) activated quickly, with time constants (fitted from 20% to peak) of 0.13 ± 0.04 (*n* = 6) and 0.2 ± 0.1 ms (*n* = 5; *P* = 0.057, two‐tailed randomisation test; Table 4, data plotted in Fig. 6*C*). Given that the 20–80% solution exchange time was on average 0.12 ± 0.04 ms (*n* = 25 patches), these rise times are the expression of the solution exchange, rather than the receptor response. The deactivation phase after the end of the pulse for homomeric and heteromeric GlyRs had time constants of 12 ± 5 and 10 ± 6 ms, respectively (fitted from 80 to 20% of peak with a single exponential; *n *= 7 and 5 patches; *P* = 0.51, two‐tailed randomisation test; Table 4, Fig. 6*D*). Figure 6 shows that introducing the α1S270T mutation speeded up the deactivation of both homomeric and heteromeric GlyRs in response to 3 mm glycine (black traces in Fig. 6*A* and *B*, plot in Fig. 6*D*) to 1.2 ± 0.3 and 1.5 ± 0.6 ms, respectively (*n *= 6 and 7; *P* = 0.0018 and *P* << 10^−6^, two‐tailed randomisation test, compared to wild‐type homomeric and heteromeric GlyR). The time course of the onset phase was not affected by the mutation. These results indicate that synaptic currents mediated by homomeric or heteromeric GlyR bearing the α1S270T mutation would be much shorter.

## Discussion

We studied the effects on human recombinant homomeric and heteromeric GlyRs of the naturally occurring mutation α1S270T, which is known to cause dominantly inherited startle disease/hyperekplexia in man (Lapunzina *et al*. 2003). Its functional effects have been reported to be much less severe in heteromeric GlyRs (Xiong *et al*. 2014). We show here that the function of mutant GlyR was rescued by β subunit co‐expression only in equilibrium measurements of glycine efficacy. Experiments more relevant to the conditions during synaptic transmission, such as outside‐out current responses to fast glycine concentration jumps, show that the function of GlyR was equally impaired for heteromeric and homomeric receptors, and mutants deactivated much more quickly than wild‐type channels.

### Can structural data shed light on how the α1S270T mutation impairs GlyR function?

**Figure 7:**
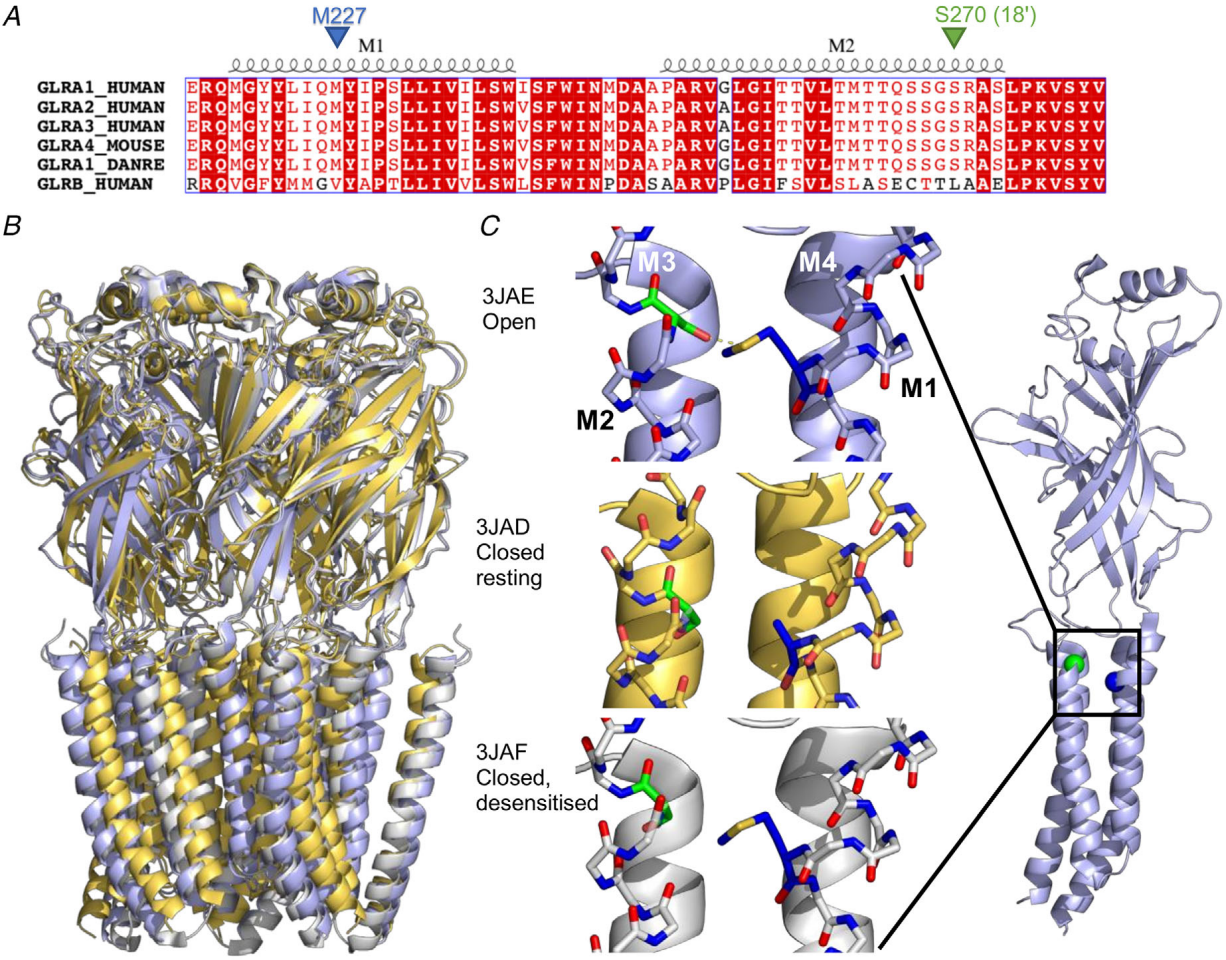
Location of the S270 (18ʹ) startle disease residue in the M2 transmembrane helix of the glycine receptor *A*, sequence alignment of the M1 and M2 transmembrane helices and the M2‐M3 linker region for the three functional human α GlyR subunits, the mouse α4 GlyR subunit, zebrafish α1 subunit and the β human GlyR subunit showing the position of S270 in M2 and that of M227 in M1. The extent of the M1 and M2 α‐helices is indicated above the alignment. Red background indicates the residue is conserved in all sequences and red font signifies residues that have similar properties. *B*, glycine receptor architecture: resting closed (yellow, Protein Data Bank code 3JAD), open (light‐blue, 3JAE) and desensitised closed structures (light‐grey, 3JAF, all from Du *et al*. 2015). *C*, the single subunit view shows (right) the position of S270 (green sphere) at the end of the M2 helix close to the extracellular side, and that of M227 (blue sphere) at the start of M1, also close to the extracellular side. This area is shown in detail in the three structures in the middle, where M1 and M2 are shown in stick format, S270 in green and M227 in blue. The M227 side chain in the open state was modelled using the mutagenesis functionality in PyMOL (The PyMOL Molecular Graphics System, Version 2.0 Schrödinger, LLC), where the rotamer closest to the one adopted by M227 in the desensitised state was chosen. The dashed line in the open state shows the hydrogen bond interaction between S270 and M227 (M227:S to S270:O distance = 2.2 Å). [Color figure can be viewed at wileyonlinelibrary.com]

Like other pentameric ligand‐gated channels, GlyRs are formed by five subunits arranged around a central pore lined by the M2 α‐helices, surrounded by the other transmembrane helices (M1, M3 and M4). The extracellular end of M2 (where S270 is) and the M2‐M3 loop are important in channel gating and many dominant hyperekplexia mutations are found in these areas (Lynch *et al*. 1997; Lewis *et al*. 1998; Bode & Lynch, 2014). S270/18ʹ (green sphere in Fig. 7*C*) is in the penultimate turn of M2, is conserved in GlyR α subunits (but not in β; Fig. 7*A*) and is not likely to be exposed to the pore (by analogy with GABA_A_ channels; Xu & Akabas, 1993). The adjacent R271 residue, the first startle mutation to be discovered (R to L/Q)(Shiang *et al*. 1993), moves during activation to stabilise the open state by interacting with Q226 (another conserved startle residue) in the M1 of the adjacent clockwise subunit (Pless *et al*. 2007; Bode & Lynch, 2013; Du *et al*. 2015). The same cryoEM structures suggest that the interactions of S270 are different, and that its side chain (green sticks in Fig. 7*C*) points *intrasubunit*, towards M3 when the channel is resting or desensitised (yellow and light grey in the figure), and towards M1 (M227, blue sticks) of the same subunit when the channel is open (light blue). The side chain of M227 is resolved only in the desensitised structure (bottom of Fig. 7*C*), but modelling it in the open structure (top of Fig. 7*C*) suggests that its sulphur atom is close enough to the hydroxyl of S270 to form a hydrogen bond (2.2 Å, dashed line in Fig. 7*C* top). If this hydrogen bond adds to the stability of the open state, anything that disrupts it would impair channel gating. The S270T startle mutation is a conservative change, but the additional methyl on the β carbon may affect the rotation kinetics of the side chain or have an impact on the interactions of the adjacent R271 residue.

These explanations are plausible, but do not suggest a straightforward mutational test of their validity. We must consider them with great caution, especially since there is still uncertainty as to the real conformation of the GlyR open channel (see the molecular dynamics proposals of different open states: Cerdan *et al*. 2018; Dämgen & Biggin, 2020).

### The effects of the S270T mutation in homomeric and heteromeric GlyR

We chose to study S270T because it has not been fully characterised before and because this mutation is of special interest for the homomeric/heteromeric pathology of startle disease, given the report that its effects are rescued by co‐expression of the GlyR β subunit (Xiong *et al*. 2014).

There are few data in the literature on the functional rescue of α subunit startle disease mutations by co‐expression of the β subunit and they are inconsistent. Rea *et al*. (2002) found no β rescue for mutation R392H expressed in oocytes, but the effects of this mutation were attributed to failure of the receptor to reach the membrane. A very small degree of β rescue was reported for R392H expressed in heteromeric GlyR in HEK293 cells (Chung *et al*. 2010).

Our own work in GlyR expressed in HEK293 showed evidence of a partial functional rescue in heteromeric GlyR for both the mouse *spasmodic* mutation (α1A52S) and the human startle α1K276E mutation. The loss of function caused by these mutations in homomers was so extreme that they could not be characterised at single channel level, whereas some functional channels were recorded for mutant heteromeric channels (Plested *et al*. 2007; Lape *et al*. 2012). Interestingly, data from our first characterisation of the K276E mutation in oocytes are in conflict with this rescue, showing an identical loss of glycine sensitivity for homomeric and heteromeric channels (Lewis *et al*. 1998).

Our present work on S270T shows that there was no β rescue at whole‐cell level and the mutation produced essentially the same shift in the dose‐response curves of homomeric and heteromeric GlyRs. We took extreme caution in repeating experiments so that all four receptors (wild‐type and mutant, homomer and heteromer) could be examined in the same batches of transfections. We also made sure that a fast exchange for the agonist applications was consistently obtained in whole‐cell U‐tube applications (0.5–1 ms). This is important, because the decay of whole‐cell current responses was quite fast in the mutants and in wild‐type heteromers (Fig. 1). This decay is likely to be due to desensitisation and will distort the measurement of peak currents, making it prone to substantial artefactual variation if the speed of the application system is not consistently optimised. Given that macroscopic dose‐response curves are distorted by desensitisation, it was particularly important to measure agonist sensitivity also at single channel level, where we can measure open probability in clusters of openings, and the long shut times caused by desensitisation are excised. In the single channel experiments on homomeric GlyRs we found a decrease in the sensitivity to glycine of about 9‐fold. This change was accompanied by a dramatic loss of channel gating, which took the maximum *P*_open_ elicited by glycine from almost 1 in wild type to 0.2 in the mutant (Table 2). This profound loss of gating transforms glycine into a partial agonist, a result that agrees with the marked reduction in the activity produced by the partial agonist sarcosine. In mutant homomer GlyRs, sarcosine failed to produce clusters of openings, eliciting a maximum *nP*_open_ value of 0.01 (Table 2). This single‐channel value is in good agreement with the whole‐cell results, where the sarcosine maximum response was 8% of that to glycine. Given that the glycine maximum *P*_open_ in the homomeric mutant was 0.16, we would expect a sarcosine *P*_open_ of 0.012, in broad agreement with the experimental *nP*_open_ estimate.

In the heteromer we saw a rescue of channel function, as the maximum equilibrium *P*_open_ was restored to 0.96, a value similar to that observed in the wild type. However, this maximum open probability was reached at much higher glycine concentrations, as glycine sensitivity was decreased by about 5‐fold in the mutant.

### Impact at the synapse of the loss of function produced by the S270T mutation

We next addressed the question of how these changes affected glycine responses produced by applications that simulate the fast, short exposure experienced by receptors at the synapse. The best estimate available (for mammalian motoneurones in spinal cord slices) indicates that the glycine transient reaches a concentration of 2.2–3.5 mm, for a fraction of a millisecond, and decays with a time constant of 0.6–0.9 ms at room temperature (Beato, 2008). We have previously shown that fast applications of millimolar glycine to wild‐type receptors in outside‐out patches evoke currents with a decay kinetics similar to that of glycinergic synaptic currents in spinal cord slices, provided the correct intracellular chloride concentration is used in the recordings (Pitt *et al*. 2008). Our data here show that currents elicited with 2 ms pulses of 3 mm glycine decayed much more quickly for mutant GlyRs. The time constant of decay was speeded up by approximately 6‐fold for both homomers and heteromers, from the wild‐type values of 12 and 10 ms to 1.2 and 1.5 ms, respectively.

**Figure A1:**
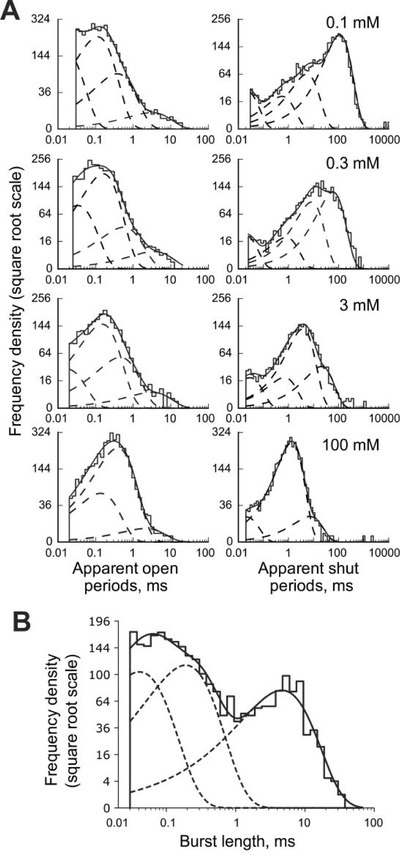
Open and shut time distributions for homomeric α1S270T glycine channels *A*, Histograms (stepwise lines) of open (left column) and shut (right column) times fitted with mixed exponential densities are shown for 0.3, 3 and 100 mM glycine. Fits are shown as solid lines and single components are shown as dotted lines. For the results of fits see Appendix Table A1. Four exponential components were used for both open and shut times at concentrations up to 3 mM and three at higher concentrations. Note that apparent shut times shortened with increasing glycine concentration. Unlike in wild‐type receptors, open time distributions retained more than one exponential component at high glycine concentrations. *B*, The experimental distribution of burst length of openings of homomeric α1S270T GlyR at 100 μM glycine (*t*_crit _ =  9.2 ms, resolution 30 μs) was fit with three exponential components: τ_1_ = 41 μs (area 35.1%), τ_2_ = 190 μs (19.0%), and τ_3_ = 4.6 ms (26.2%).

The time constant of macroscopic current decay after the agonist concentration is ‘jumped’ to zero is known to contain the same time constants as the burst distributions in single channel records at steady state (Wyllie *et al*. 1998). Despite the difficulty in analysing the mutant single channel records, we verified the effects of the mutation on the burst time distributions on homomeric GlyRs, which were the mutant channels that were least heterogeneous and lent themselves better to single channel analysis (Appendix Fig. A1). Here we found a shorter burst length in agreement with that observed in macroscopic concentration jumps onto outside‐out patches, further confirmation that synapses where mutant receptors are expressed would suffer a substantial decrease in charge transfer.

Some questions remain. First of all, the mutation produces a dominant form of startle disease and the patients’ GlyRs will contain a mixture of mutant and wild‐type α subunits. Thus, it is to be expected that the effects of the mutation may be attenuated in the channels *in vivo*, to an extent that we cannot predict from our experiments. However, charge transfer will be affected also by the amplitude of the synaptic current. A decrease in peak amplitude is plausible, given the decreased sensitivity to glycine in the heteromer. Neither the whole‐cell nor the jump experiments contain the information we need, i.e. the peak open probability reached in response to the millisecond rise in glycine concentration at the synapse, because we do not know the number of channels in each recording. A robust calculation of this non‐equilibrium peak open probability requires an adequate kinetic model of the mutant channel. We could not achieve this even for homomeric mutant channels (data not shown), probably because we could analyse only high glycine concentrations (where we could check that we had the dominant mode of activity in the clusters) and because the presence of more than one open state at high glycine concentrations made it necessary to fit a complex activation mechanism.

Finally, given that we could not carry out global mechanism fits to the mutant single channel data, can we nevertheless identify which of the steps in receptor activation is likely to be most affected by the mutation? We systematically explored how changes in the values of each rate constant in the wild‐type GlyR activation mechanisms affected glycine EC_50_, equilibrium maximum *P*_open_ and deactivation time constants (see Appendix). Impairing the first gating step (the transition to the pre‐open ‘flip’ intermediate state, Burzomato *et al*. 2004) failed to account for the changes in deactivation we observed and could be excluded. On the other hand, impairing channel opening by decreasing the opening rate constant predicted effects that approximated well those observed for mutant homomeric GlyRs. However, the simple change we modelled (only one rate constant, similar changes at all levels of ligation) accounted only in part for the mutation effects seen in heteromeric GlyRs. It is likely that combining changes in opening and closing rate constants, or changing only the fully liganded rate constants would improve predictions, but this implies exploring a large parameter space with limited constraints by the data.

Our experiments show that the α1S270 mutation causes substantial loss of function in both homomeric and heteromeric GlyRs. This conclusion is strengthened by the good agreement between data obtained with different patch‐clamp recording modes and with different types of agonist applications, especially those that mimic synaptic conditions.

## Additional information

### Competing interests

The authors declare that they have no conflict of interests.

### Author contributions

L.G.S., R.L. and T.G. conceived and designed the experiments; Z.W., R.L., L.J.‐S., B.J.O'C. and T.G. performed and analysed experiments; Z.W., R.L. and L.G.S. wrote the manuscript. All authors approved the final version of the manuscript and are accountable for all aspects of the work. All persons designated as authors qualify for authorship, and all those who qualify for authorship are listed.

### Funding

This work was supported by MRC project grants, MR/J007110/1 to L. Sivilotti and P. Biggin and MR/R009074/1 to L. Sivilotti.

## Supporting information


**Statistical Summary Document**

## Figures and Tables

**Figure A2:**
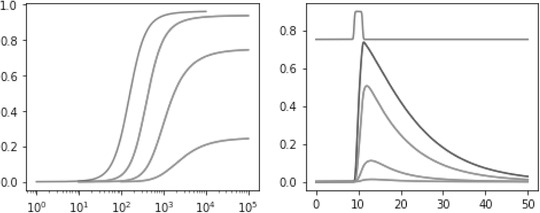
Homomeric GlyR: Effect on GlyR concentration‐ *P*_open_ curves (left) and on the macroscopic current response to a 2 ms pulse of 3 mM glycine (right) of reducing all three forward flipping rates, δ1, δ2 and δ3 by 10, 100 or 1000‐fold. With the greatest change (1000‐fold), the max*P*_open_ dropped down from 0.96 to 0.25, the *EC*_50_ increased by 16‐fold and the deactivation τ decreased by 2‐fold (14 ms to 6.7 ms). Note that the change in *EC*_50_ is larger than the one we observed and that deactivation did not become as fast as the one observed in experiments. Similar effects were seen for heteromeric GlyR (results not shown).

**Figure A3:**
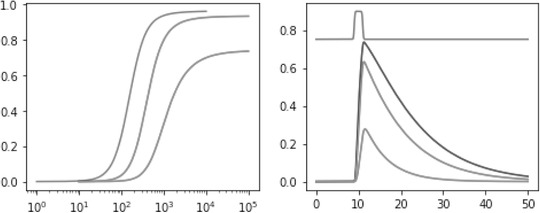
Homomeric GlyR: Effect on GlyR concentration‐ *P*_open_ curves (left) and on the macroscopic current response to a 2 ms pulse of 3 mM glycine (right) of increasing all three backward flipping rates, γ1, γ2 and γ3, by 10 or 100‐fold. With a 100‐fold increase, max*P*_open_ dropped down from 0.96 to 0.74, *EC*_50_ increased by 7.3‐fold, and deactivation τ decreased from 14 ms to 5.6 ms. Note that again deactivation did not become as fast as the one observed in experiments. We could not increase the values of γ’s more than 100‐fold because the value of the fully liganded γ became too high to be realistic (>>1,000,000 per second). Similar effects were seen for heteromeric GlyR (results not shown).

**Figure A4:**
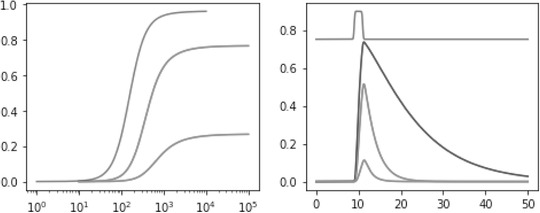
Homomeric GlyR: reducing all three opening rate constants, β1, β2 and β3, by 10 or 100‐fold affected both max*P*_open_ and macroscopic current decay. max*P*_open_ dropped down from 0.96 to 0.27, *EC*_50_ increased by 4.9‐fold and deactivation τ decreased from 14 ms to 1.6 ms when all β’s were reduced by 100‐fold. Note that the *EC*_50_ change is similar to that observed and so is deactivation, which did become as fast as the one observed in experiments.

**Figure A5:**
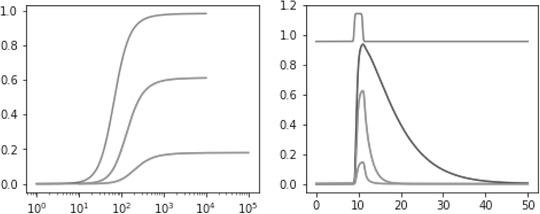
Heteromeric GlyR: reducing all three opening rate constants, β1, β2 and β3, 10 or 100‐fold affected both max*P*_open_ and macroscopic current decay. max*P*_open_ dropped down from 0.98 to 0.18, *EC*_50_ increased by 3.3‐fold, and deactivation τ decreased from 10 ms to 0.6 ms when all β’s reduced 100‐fold, values that are comparable to those observed. However, the decrease in max*P*_open_ is much larger than the one we observed experimentally.

**Figure A6:**
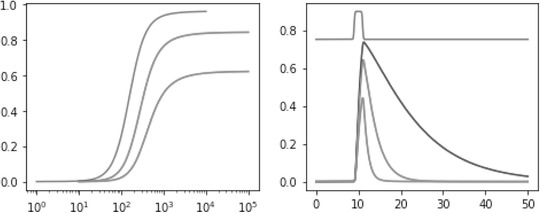
Homomeric GlyR: increasing all closing rate constants, α1, α2 and α3 by 5‐ or 20‐fold. max*P*_open_ dropped down from 0.96 to 0.62, *EC*_50_ change 2.9x, and deactivation τ decreased from 14 ms to 1.0 ms when all α’s were increased by 20‐fold.

**Figure A7:**
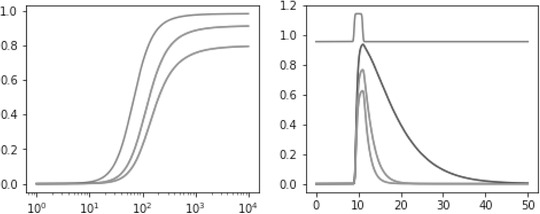
Heteromeric GlyR: increasing all closing rate constants, α1, α2 and α3, 5‐ or 10‐fold. max*P*_open_ dropped down from 0.98 to 0.80, *EC*_50_ change 2.3x and deactivation τ decreased from 10 ms to 1.3 ms when all α’s increased 10‐fold.

## Data Availability

The data that support the findings of this study are available from the corresponding author upon reasonable request.

## Dwell‐time distributions of openings, shuttings and bursts for homomeric mutant receptors

Analysing in detail the behaviour of mutant GlyRs at single channel level is made more difficult by the heterogeneity we observed especially in the heteromeric records. However, in homomeric α1S270T GlyRs, there is a clear dominant low *P*_open_ mode, which can be distinguished by an independent criterion, eg because of its shorter open times. We attempted to extract a consistent set of homomeric data by discarding the relatively few clusters which had mean open time longer than 1 ms.

Appendix Figure A1*A* shows dwell‐time distributions from 4 cell‐attached homomeric recordings. The apparent open period plots in the left column and the shut time plots in the right column were obtained from single channel records idealised by time‐course fitting. The solid curves show fits with mixtures of exponential probability density functions, and each component is shown as a dashed curve. Fitted parameters are summarised in Appendix Table A1. Four components were necessary to fit well the open period distributions at 0.1‐3 mM glycine and even at the highest two glycine concentrations (10 and 100 mM) three components were required. The complexity of the open period distribution was rather surprising given that the open‐period distribution of wild‐type homomeric GlyRs is mono‐exponential at saturating glycine (Beato *et al*., 2004). In contrast, open‐period distributions of the α1S270T mutant retained at least three exponential components even at the highest agonist concentration tested (100 mM), suggesting that more than one open state is accessible when receptors are fully‐liganded. The time constants of the three longest open components remained relatively stable in the range of glycine concentrations tested (0.1‐100 mM). At the concentrations where four components can be clearly distinguished, the time constant of the fastest components was also relatively stable, but its area dropped from 39% at 0.1 mM to 16% at 3 mM. This component is likely to be still present at glycine concentrations 10 mM and higher, but more difficult to resolve, as its area is very small. At the highest concentration (100 mM) the predominant open component time constant was 0.39 ms, the second longest.

The shut time distributions required four exponential components for an adequate fit, except at the two highest concentrations tested (10, 100 mM; Appendix Figure A1*A* right column and Appendix Table A1). As the glycine concentration increased from 0.1 mM to 3 mM, the slowest component decreased in area from 58% to 11%, and could not be reliably detected at concentrations higher than 3 mM. The time constants of other, faster shuttings remained relatively stable between 0.1 mM and 100 mM. Interestingly, the fastest shut time component, which is dominant in the wild‐type homomeric receptor shut time distribution at saturating glycine (1 mM; Beato *et al*., 2004) had a relatively small area at saturating glycine concentration in the homomeric mutant. The predominant shut time component at 100 mM was the second fastest, with a time constant of about 1 ms.

Both synaptic currents and current relaxations to agonist concentration‐jumps are non‐equilibrium phenomena, but it is well established that the time constants of deactivation are the same as in low agonist concentrations, steady‐state single channel recordings analysed into bursts (the areas of the components are different, Wyllie *et al*., 1998). Bursts (groups of openings between the binding and the dissociation of the agonist) are identified because they are separated by shut intervals which are concentration dependent because they are ended by the binding of the agonist.

Because of the heterogeneity in heteromeric records, we attempted burst analysis only in data from homomeric mutant α1S270T GlyR. These channels had complex shut time distributions (Appendix Figure A1*B*), with several partially overlapping components and it was unclear whether only the longest shut time or the two longest shut times were concentration‐dependent (see Appendix Table A1). We analysed two patches at the lowest glycine concentration (0.1 mM), empirically setting the critical time interval between the second and the third or between the third and the fourth shut time components (see the right column in Appendix Figure A1). In the first case, the burst distribution resembled the open time distribution, i.e. bursts had one opening, with average duration is 0.266 and 0.295 ms in the two patches. The second choice of critical time interval produced a burst distribution with three components (Appendix Figure A1*B*). In the two patches, bursts contained an average 1.5 openings and had an average duration of 1.7 and 2.3 ms. Such short time constants for single channel burst length (around 2 ms) are in qualitative agreement with the speeding of the deactivation observed in the concentration jumps. These data show that the α1S270T mutation shortens the time GlyR receptors stay open, measured both at single channel level (in cell‐attached patches) and in response to quasi‐synaptic agonist applications (to outside‐out patches). This confirms that synaptic currents mediated by mutant receptors would be much shorter.

**Table A1:** Time constants and areas of the components of open‐ and shut‐time distributions at different glycine concentrations for the dominant mode of homomeric α1S270T GlyR

Glycine, mM (*n* of patches)	τ1, ms (area, %)	τ2, ms (area, %)	τ3, ms (area, %)	τ4, ms (area, %)
Open time				
0.1	0.015 ± 0.02	0.113 ± 0.004	0.38 ± 0.02	3.1 ± 0.2
(2)	(39 ± 3)	(46 ± 1)	(14 ± 2)	(1.49 ± 0.01)
0.3	0.033 ± n/a	0.159 ± 0.005	0.56 ± 0.02	3.53 ± 0.06
(2)	(33 ± 4)	(54 ± 4)	(11.46 ± 0.03)	(2.15 ± 0.09)
1	0.014 ± 0.008	0.098 ± 0.06	0.34 ± 0.07	2.5 ± 0.5
(2)	(31 ± 7)	(51 ± 5)	(16 ± 2)	(2 ± 0.2)
3	0.016 ± 0.002	0.149 ± 0.005	0.45 ± 0.01	3.2 ± 0.1
(3)	(16 ± 5)	(47 ± 6)	(34 ± 6)	(3 ± 0.4)
10		0.099 ± 0.006	0.29 ± 0.01	2.4 ± 0.2
(3)		(50 ± 2)	(44 ± 3)	(5.5 ± 0.7)
100		0.135 ± 0.004	0.385 ± 0.005	2.1 (SD not defined)
(3)		(30 ± 10)	(67 ± 10)	(3 ± 0.7)
Shut time				
0.1	0.016 ± 0.005	0.6 ± 0.2	5.6 ± 0.1	120 ± 10
(2)	(17 ± 5)	(7 ± 1)	(17.9 ± 0.1)	(58 ± 4)
0.3	0.021 ± 0.006	1.4 ± 0.4	14 ± 2	100 ± 20
(2)	(16 ± 6)	(12 ± 3)	(37 ± 3)	(35.1 ± 0.1)
1	0.014 ± 0.003	2.6 ± 0.4	26 ± 2	120 ± 10
(2)	(14.8 ± 0.7)	(18.2 ± 0.6)	(53 ± 5)	(14 ± 4)
3	0.028 ± 0.008	0.8 ± 0.1	4.2 ± 0.5	22 ± 2
(3)	(15 ± 3)	(29 ± 4)	(45 ± 4)	(11 ± 4)
10	0.2 ± 0.1	1.8 ± 0.2	9 ± 1	
(3)	(15 ± 5)	(69 ± 5)	(15 ± 1)	
100	0.019 ± 0.02	1.28 ± 0.05	6.2 ± 0.5	
(3)	(9 ± 3)	(75 ± 5)	(17 ± 4)	

Note that for open period distributions, data from all patches at the same concentration were fitted simultaneously, constraining the time constant values to be the same. Hence the approximate standard deviation displayed in this table was calculated from the covariance matrix.

### Modelling channel activation to identify the step affected by the S270T mutation

The main observations that we need to account for in mutant vs wild type (WT) GlyRs were:

1. decreased sensitivity to glycine (both homomeric and heteromeric receptors, 4‐fold and 3‐fold respectively)
2. decrease of the maximum open probability (*P*_open_) at equilibrium. This decrease is pronounced in homomeric GlyRs, but very small in heteromeric GlyRs (from 99% WT for both to 16% and 91%, respectively, see table 3).
3. speeding up of the deactivation of currents evoked by concentration jumps in both homomeric and heteromeric receptors (10‐fold and 7‐fold, respectively). Note that the *activation* time constant of wild type GlyR currents is determined by the solution exchange time constant, so the real (receptor) time constant cannot be measured, even with our 0.12 ms exchange time constant.

#### Maximum *P*_open_ at equilibrium

Observation 2) shows that there is a decrease in maximum *P*_open_, particularly for homomeric GlyRs (this is confirmed also by the reduction in the relative maximum response to sarcosine), and this indicates that the mutation impairs gating.

In the best established model for the activation of GlyRs, the ‘flip’ mechanism (Burzomato et al., 2004; Lape et al., 2008), the maximum *P*_open_ at equilibrium depends on two gating steps, access to the intermediate flip state and opening. Gating can be impaired by reducing the efficiency of either step. Thus

$$\;maxP_{open}=\frac{FE}{FE+F+1}\;$$

where F and E are the equilibrium constants in the two gating steps for the fully liganded receptor

#### Modelling of mutation effects

We calculated what magnitude of changes in each of the rate constants associated with the two gating steps (cf. the values we estimated for homomeric and heteromeric GlyRs in Burzomato *et al*., 2004) would lead to maximum *P*_open_ values compatible with those observed for the mutants. Then we checked what the effects of these rate constant changes would be on the glycine *EC*_50_ and on the macroscopic deactivation time constant.

Wild‐type mechanism and rate constant values were taken from Tables 6 (homomeric GlyR) and 5 (heteromeric GlyR) in Burzomato *et al*., J. Neurosci. 24 (2004), 10924. Equilibrium *P*_open_ curves and macroscopic currents were calculated using the SCALCS package (available at https://github.com/DCPROGS/SCALCS).

Macroscopic currents were calculated as responses to a realistic profile of solution exchange for a 2 ms, 3 mM glycine pulse (2 ms halfwidth with symmetrical 0.2 ms 10–90% rise and fall in concentration). Deactivation τ values were obtained by fitting one exponential to the calculated current decay phase. The fit was done in Clampfit 10.7 (Molecular Devices).

#### Changes in flipping rate constants

*Changes in entry or exit to the flip intermediate state are unlikely to mediate the effects of the mutation because they cannot account for the speeding in macroscopic deactivation*. This is shown in Appendix Figures A2 and A3.

The examples show in detail the effects on the glycine concentration *P*_open_ curves (left) and on the time course of macroscopic current responses to saturating glycine (right) of reducing all the three forward flipping rates (δ, Appendix Figure A2) or increasing all the three backward flipping rates (γ, Appendix Figure A3). In each case the results of calculations with the WT rate constants are shown in blue and the results of specific changes in rate constants in red. For detailed comments, see the legends.

Slowing entry into flip (δ rate constant) by up to 1000‐fold (to decrease the maximum *P*_open_ sufficiently) produced only a modest change in the deactivation of macroscopic glycine currents. In addition to that, sensitivity to glycine was reduced by more than we observed in our experiments. Similar findings were obtained changing the exit from the flip state (γ). Note that γ could not be speeded up by more than 100‐fold because greater changes gave γ a value much greater than 1,000,000 per second, which is not realistic for a protein conformational change.

#### Changes in opening rate constants

*Impairing channel opening, by decreasing the opening rate constant or speeding the closing rate constant, predicted effects that approximated well those observed for mutant homomeric GlyR. The simple change we modelled (only one rate constant, similar changes at all levels of ligation) accounted only in part for the mutation effects seen in heteromeric GlyRs. It is likely that combining changes in opening and closing rate constants, or changing only the fully liganded rate constants would improve predictions, but this implies exploring a large parameter space*. This is shown in Appendix Figures A4, A5, A6, A7.

We explored first the effects of reducing the opening rate constants β (at all levels of ligation) by up to 100‐fold. We found that this change had similar effects on homomeric and heteromeric receptors: it reduced both glycine sensitivity and maximum *P*_open_ and speeded up deactivation. The greatest reduction in β produced changes in glycine sensitivity and deactivation that were comparable to those observed experimentally. This also predicted a greatly reduced maximum *P*_open_ for both receptor types. This was similar to our observations for homomeric receptors, but not for heteromeric GlyR.

The alternative way to impair the opening step is increasing the rate of closing (α). Increasing α (at all levels of ligation) affected both glycine sensitivity and deactivation to an extent that was similar to that observed in our experiments. However, the effects predicted on max*P*_open_ were too small cf. our observations in homomers.

The good prediction of some of the mutant channel behaviours and the asymmetry in the effects on max*P*_open_ of changing either opening or closing rate constants suggest that modelling a combination of effects on both rate constants could be successful. An additional possibility would be to allow rate constants to vary independently at all levels of ligation. This makes sense in principle for heteromers, where two of the three binding interfaces needed for full function are formed by different subunits (α subunit/β subunit) and the third is formed by the same subunit (α subunit/α subunit) and could be more deeply affected by an α subunit mutation.

Exploring all these possibilities would not be a sensible option, given that we have limited experimental data to constrain the parameter space.
