# Supplementary material for: The startle disease mutation α1S270T predicts shortening of glycinergic synaptic currents
Source: J Physiol. 2020 Jun 18;598(16):3417–38. doi: 10.1113/JP279803 (PMC7649747; doi:10.1113/JP279803)
Supplement: Supplementary file 1 — Statistical Summary Document [file TJP-598-3417-s001.docx]

**Manuscript Title: *The Startle Disease mutation, α1S270T, predicts shortening of glycinergic synaptic currents***

**Authors:** *Zhiyi Wu, Remigijus Lape, Lea Jopp-Saile, Benjamin J. O’Callaghan, Timo Greiner and Lucia G. Sivilotti*

**Animal model used, if applicable:** not applicable

**Underlying hypothesis:** This investigation tests the hypothesis that α1S270T mutation affects glycine receptor sensitivity to agonists, single channel open probability and synaptic response.

**Definitions of ‘n’:**

Question 1: n = number of individual concentration-response curves obtained from multiple cells.

Question 2: n = number of single-channel activity clusters recorded from multiple cells.

Question 3: n = number of outside-out patches.

**Statistical summary table:**

| Experimental question number* | Finding/ conclusion | Experimental location/ variable  e.g. cortex vs cerebellum or genotype | Mean value  (or other summary statistic) | SD | n (value) | P** | Units | Data comparisons  e.g. WT vs KO | Statistical test | Any other variable  e.g. subjects’ age or sex | Figure/table in which data are presented | Comments  e.g. observation |
| --- | --- | --- | --- | --- | --- | --- | --- | --- | --- | --- | --- | --- |
| Question 1: Concentration-response curve *EC*_50_? | α1S270T mutation increases *EC*_50_ | Glycine, WT α1 | 0.24 | 0.06 | 10 | - | mM | - | Randomisation test |  | Figure 1  Table 1 | Hill equation fits to agonist concentration-response data from whole-cell recordings; holding potential −60 mV, internal chloride 30 mM. |
|  |  | Glycine, WT α1β | 0.23 | 0.03 | 5 | 0.93 | mM | Vs Glycine, WT α1 |  |  |  |  |
|  |  | Glycine, α1S270T | 1.1 | 0.2 | 8 | 0 | mM | vs Glycine, WT α1 |  |  |  |  |
|  |  | Glycine, α1S270Tβ | 0.9 | 0.1 | 8 | 0.00034 | mM | vs Glycine, WT α1β |  |  |  |  |
|  |  | Sarcosine, WT α1 | 14 | 6 | 7 | 0.00004 | mM | vs Glycine, WT α1 |  |  | Figure 3  Table 1 |  |
|  |  | Sarcosine, α1S270T | 23 | 3 | 6 | 0.0076 | mM | vs Sarcosine, WT α1 |  |  |  |  |
| Question 2:  Single-channel maximal open probability? | α1S270T mutation reduces maximal open probability in homomeric but not heteromeric receptor | 10 mM Glycine, WT α1 | 0.99 | 0.02 | 45 | - |  | - | Randomisation test |  | Figure 2  Table 2 | Cell-attached single channel recordings; Vcomm= +100 mV; open/shut transitions detected using half-amplitude threshold algorithm. |
|  |  | 10 mM Glycine, WT α1β | 0.993 | 0.006 | 87 | 0.0018 |  | vs Glycine, WT α1 |  |  |  |  |
|  |  | 100 mM Glycine, α1S270T | 0.16 | 0.1 | 128 | 0 |  | vs Glycine, WT α1 |  |  |  |  |
|  |  | 100 mM Glycine, α1S270Tβ | 0.96 | 0.03 | 131 | 0 |  | vs Glycine, WT α1β |  |  |  |  |
| Question 3:  Macroscopic current deactivation time constant? | α1S270T mutation speeds up deactivation | WT α1 | 0.13 | 0.04 | 6 | - | ms | - | Randomisation test |  | Figure 6  Table 4 | 2 ms pulses of 3 mM glycine to outside-out patches. V holding −111 mV; 30 mM internal Cl^−^. |
|  |  | WT α1β | 0.2 | 0.1 | 5 | 0.51 | ms | vs WT α1 |  |  |  |  |
|  |  | α1S270T | 0.4 | 0.1 | 6 | 0.0018 | ms | vs WT α1 |  |  |  |  |
|  |  | α1S270Tβ | 0.18 | 0.03 | 7 | 0 | ms | vs WT α1β |  |  |  |  |

*You may use multiple lines for the same question to indicate multiple comparisons

** Authors may wish to make the text bold where p is considered significant against a stated confidence limit
